# The Identification of Six Estrogen Preparations by Combining Thin-Layer Chromatography with Micro-Raman Imaging Spectroscopy

**DOI:** 10.3390/molecules29225328

**Published:** 2024-11-12

**Authors:** Wenquan Zhu, Xin Liang, Mengjiao Li, Xinrui Wang, Hongxia Cui, Yan Dong, Hongzhou Bu, Wei Dong, Huimin Sui, Feng Xu, Yuhui Fang, Chunhui Ma

**Affiliations:** 1 School of Pharmacy, Qiqihar Medical University, Qiqihar 161006, China; zhuwenquan1984@126.com (W.Z.); liangxin@qmu.edu.cn (X.L.); lmj17835844822@outlook.com (M.L.); wxrwy99@163.com (X.W.); xutianfang@sohu.com (H.C.); pingguoweiweiwei@126.com (W.D.); suihm_9@163.com (H.S.); 15845205504@163.com (F.X.); m1c2h3@163.com (C.M.); 2 Drugs Inspection Centre of Qiqihar, Qiqihar 161006, China; 15164679819@163.com (H.B.); mutong0452@163.com (Y.F.)

**Keywords:** Raman imaging microscopy, TLC, identification, estrogen preparations

## Abstract

A method for identifying six estrogen preparations by thin-layer chromatography combined with Raman imaging microscopy (TLC-RIM) was established. An appropriate pretreatment method was adopted to extract and purify the six estrogen preparations. After that, each estrogen extraction solution was spotted on a thin-layer chromatography plate. Estriol (E3), estradiol (E2), estradiol valerate (EV), estradiol benzoate (EB), nilestriol (CEE), and ethinylestradiol (EE2) were separated by TLC, and their R_f_ value and localization were determined under a UV lamp at 254 nm, followed by the in situ enrichment of the drug component. Using a 532 nm laser as the light source, the Raman scattering spectrum of the component was directly collected by micro-Raman imaging. The R_f_ values after TLC separation of the six estrogens and their Raman spectra can, respectively, reflect differences in polarity and structure, and they are not affected by the excipients of preparation. The detection limits of the six estrogens are 0.636, 1.00, 0.687, 0.497, 0.649, and 0.626 mg/mL. Based on the intensity of the minimum characteristic peak, the stability results within 40 min showed that the RSD of each substance is 1.34, 2.06, 1.65, 3.99, 1.16, and 2.71%, respectively. This method has strong specificity, good stability, and high sensitivity, and it can provide a new reference for improving the identification standards of estrogen preparations.

## 1. Introduction

**Figure 1:**
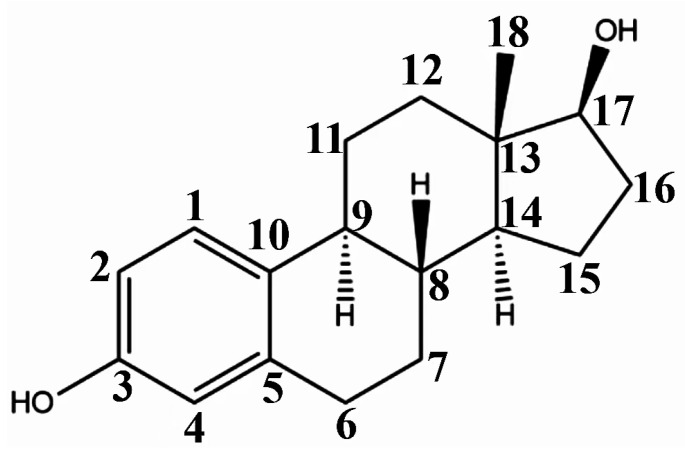
Chemical structure of estradiol (E2).

**Figure 2:**
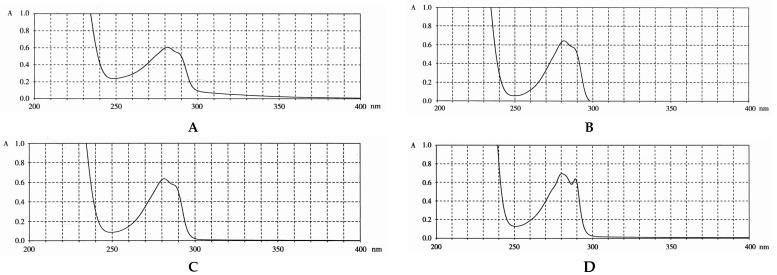
Ultraviolet spectra of estrogens ((**A**) estriol; (**B**) estradiol; (**C**) estradiol valerate; (**D**) nilestriol).

Steroid estrogens are widely employed to supplement estrogen or treat diseases caused by insufficient estrogen in women [1,2,3]. Estradiol (E2) has the strongest biological activity. There are two hydroxyl groups at positions 3 and 17 of its steroid nucleus (Figure 1.). Estradiol valerate (EV) is the esterification product of the 17β-hydroxyl of estradiol and valeric acid. Estradiol benzoate (EB) is formed by the esterification of the 3-hydroxyl of estradiol and benzoic acid. Ethinyl estradiol (EE2) is obtained by introducing an ethynyl group at the 17α position of estradiol. Estriol (E3) has an additional hydroxyl group at the 16α position based on estradiol. Nilestriol (CEE) is formed by introducing a cyclopentyloxy structure to form an ether at position 3 of estriol and also by introducing an ethynyl group at position 17. These six estrogens are widely used in clinical practice, and accurate identification is crucial. They all have the same steroid nucleus structure, and their differences are only in the substituents at C3, C16, and C17. Moreover, these drugs have diverse dosage forms, which makes it difficult for old methods to simply and quickly identify various preparation types of these drugs. This is one of the major challenges faced by researchers. The current edition of the *Chinese Pharmacopoeia* (ChP2020) contains more than 20 types of estrogen preparations, such as tablets, injections, soft capsules, and others [4]. For sustained-release estradiol tablets, chemical methods and chromatographic methods (TLC/HPLC) are used for identification. For estradiol valerate injections, chromatographic methods (TLC/HPLC) are used. For estradiol benzoate injections, chromatographic methods (TLC/HPLC) are used. For ethinyl estradiol tablets, chemical methods and chromatographic methods (HPLC) are used. Only for nilestriol tablets are chemical methods and ultraviolet spectroscopy (with maximum absorption at wavelengths of 280 nm and 288 nm) used, and this is the only preparation identified by spectroscopic methods. However, when we consult the ultraviolet spectra of these six compounds, we find that most of them are very similar (Figure 2) [5]. It is hard to say whether this method has high specificity. In the *United States Pharmacopoeia* (USP-NF), thin-layer chromatography is used to identify estradiol tablets and ethinyl estradiol tablets, while only chemical methods are specified for identifying estradiol valerate injections [6], limiting the specificity. Obviously, the most widely used method in the identification of such drug preparations at present is chromatography. However, relying solely on chromatography and close retention times [7] makes it challenging to achieve a satisfactory identification, let alone obtaining the structural information of substances. In practical work, when identifying drugs, spectroscopic methods with stronger specificity, richer structural information, and more intuitive results are more popular. However, it is not difficult to find from the pharmacopoeias of the two countries that infrared spectroscopy is only used for the identification of bulk drugs and that it cannot be used for the identification of preparations. The simultaneous use of IR and HPLC for identification can enhance specificity. However, pretreatment is complicated, a large amount of reference substances is required, and a large amount of organic solvents is consumed. The combination of the two methods is not only difficult to operate and has high test costs, but it also pollutes the environment and is not easy to popularize and use. Therefore, it is not suitable for the identification of drug preparations.

TLC is a simple and rapid separation technology for trace components. Commonly, water-containing silica gel G is used as the stationary phase [8,9,10,11]. IR is the result of changes in molecular dipole moments. Water and silica gel strongly interfere with the analysis of the measured substance and are not easily combined with TLC [12,13,14,15]. However, the Raman spectrum is the result of changes in molecular polarizability. Water and silica gel have almost no interference with this analysis. Samples can be simply processed and then separated by TLC [16,17,18,19,20,21,22], and the Raman spectra of the samples can be obtained.

This article aims to enhance the specificity of existing identification methods for steroid estrogen drug preparations. Six representative estrogen drugs were selected. First, the TLC method was initially used to separate the active ingredients, followed by in situ enrichment of the components. Then, the Raman spectrum was directly measured. Finally, by comparing with the corresponding reference standard spectrum and combined with the R_f_ value, this approach can serve as an identification method for the preparations, significantly improving the specificity of identification. This method has been widely used in the detection of illegally added substances in drugs [23,24,25]. In the study, this approach is applied to the identification of drug preparations for the first time, providing a new approach for their identification.

## 2. Results and Discussion

### 2.1. Separation of Estrogens by TLC

**Figure 3:**
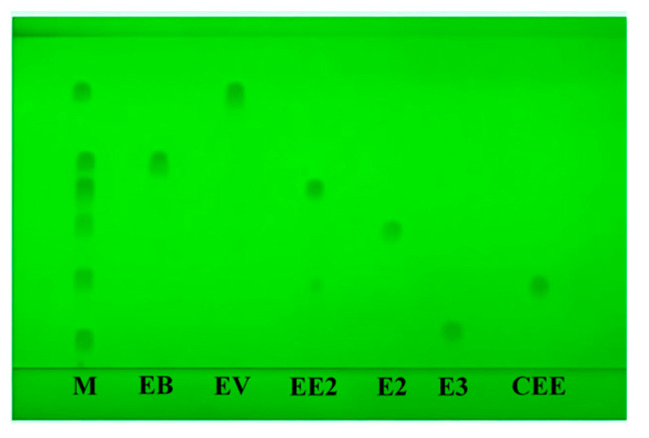
The thin-layer chromatogram of the six estrogens (M: mixture reference solution; EB: estradiol benzoate; EV: estradiol valerate; EE2: ethinylestradiol; E2: estradiol; E3: estriol; CEE: nilestriol).

The separation of E3, E2, EV, EB, CEE, and EE2 reference solution on TLC is shown in Figure 3. The R_f_ values of the main spots are 0.20, 0.41, 0.83, 0.60, 0.30, and 0.54, respectively.

### 2.2. Correlation Between Raman Spectra of Estrogens Reference Standards and the Spectra by TLC-RIM

**Figure 4:**
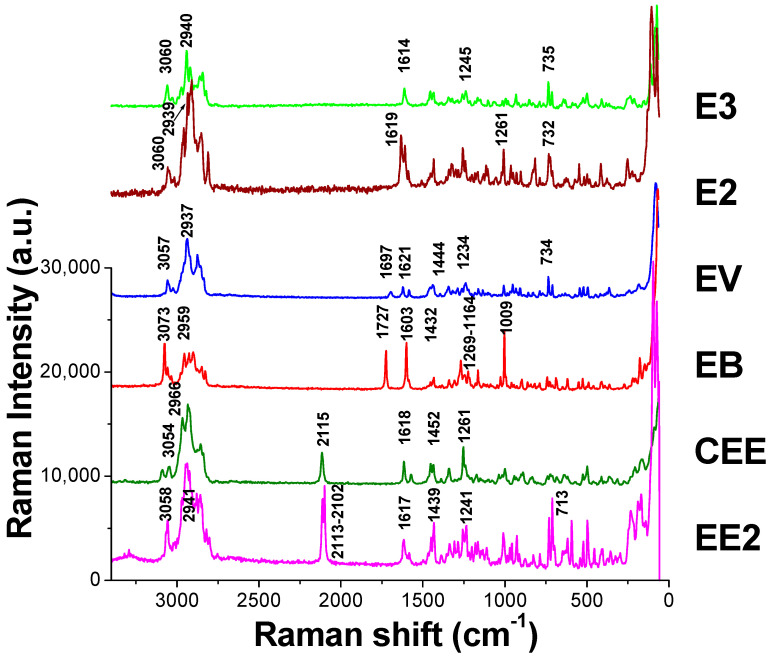
The Raman reference standards spectra of six estrogens.

**Figure 5:**
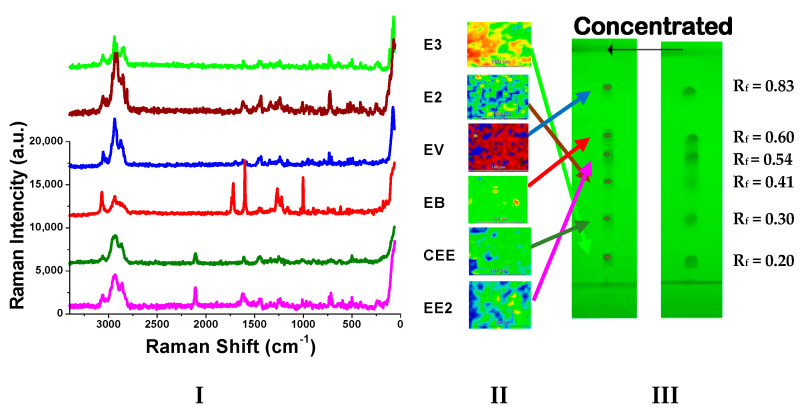
The Raman spectra from six estrogens on TLC (**I**: Raman spectra; **II**: Raman imaging; **III**: TLC plates).

The Raman spectra of reference standards are presented in Figure 4, while those after TLC separation are shown in Figure 5I. The Raman shifts, peak shapes, and relative peak intensities (with the characteristic peak from ν_=CH_ within phenyl rings as the reference peak and the intensity of other characteristic peaks divided by the reference peak) of the six main characteristic peaks of estrogens as well as their functional group assignments are listed in Table 1. Taking EE2 as an example, the similarities and differences between its spectra in Figure 4 and Figure 5 were compared. In combination with the measurement data in Table 1, the results demonstrated that the Raman shift and peak shape of the main characteristic peak were basically in agreement with each other, and there was only a slight alteration in the relative intensity of the characteristic peaks. For instance, Raman shifts of the two characteristic peaks from ν_C≡C_ emerged at 2113 cm^−1^ and 2102 cm^−1^ in the Raman spectrum of the EE2 reference standard. However, in the spectrum obtained by TLC-RIM, there was only one peak from ν_C≡C_ that appeared at 2111 cm^−1^ and a characteristic peak at 2102 cm^−1^ that nearly vanished. This might be attributed to the change in the crystal form of EE2 after being dissolved in ethanol and undergoing chromatography with the developing agent. Similarly, when comparing the Raman spectra of the other five estrogens, there were also phenomena where the intensity of individual characteristic peaks (not the main characteristic peaks) changed or disappeared, and their Raman shifts are marked in red in Table 1.

In summary, based on the Raman spectra of the six estrogens separated by TLC, except for a few characteristic peaks whose intensity had changed, most of the characteristic peaks are consistent with their standard spectra. Therefore, the Raman spectra of the six estrogens separated by TLC are consistent with their standard spectra. The spectrum has similar specificity and can also reflect the fingerprint structure information of a drug, which can be used as the basis for its identification.

### 2.3. Similarities and Differences of the Six Estrogens in Raman Spectra

The steroid core of the estrogens is composed of four rings, as shown in Table 1. Based on the substitutions, the six drugs can be divided into three categories: those containing neither carbonyl nor alkynyl, those containing carbonyl but no alkynyl, and those containing alkynyl but no carbonyl. The presence of characteristic peaks from ν_C≡C_ (2115 to 2102 cm^−1^) or ν_C=O_ (1727 to 1697 cm^−1^) in the Raman spectrum can serve as the basis for the identification of these three kinds of drugs, as depicted in Figure 4.

E3 and E2 belong to the first category since they have neither alkynyl nor carbonyl in their chemical structures. The peak intensity ratio of ν_asCH2_ (2913 cm^−1^) to ν_asCH3_ (2939 cm^−1^) in the E2 spectrum is 4.2/3.9 (value > 1). Compared with E2, E3 has one more hydroxyl group and one less CH_2_ in the five-membered ring, resulting in a decrease in the peak intensity ratio (ν_asCH2_ at 2913 cm^−1^ to ν_asCH3_ at 2940 cm^−1^) to 1.7/2.4 (value < 1). The other characteristic peaks in the spectra are almost the same, which enables the distinction between E3 and E2.

Moreover, EV and EB are classified into the second category. Their chemical structures do not contain an alkynyl group, but both contain a carbonyl group. Prominent peaks are observed at 1697 cm^−1^ for EV and 1727 cm^−1^ for EB. This pertains to the carbonyl group (C=O), which is usually experimentally observed within the 1720–1740 cm^−1^ range for ketones; due to intramolecular hydrogen bonding in EV, the absorption of the carbonyl group (C=O) appears around 1690 cm^−1^ [26] (P. 133). Additionally, the hydroxyl group in the five-membered ring of the EV forms an ester with valeric acid (fatty acid), so one ν_CH3_ and three ν_CH2_ are added to the parent nucleus. As a result, the peak intensities of EV spectra for ν_asCH2_ (2921 cm^−1^) and ν_asCH3_ (2937 cm^−1^) are increased to 2.3 and 3.2, respectively. In contrast, the phenolic hydroxyl group in EB forms esters with benzoic acid (aromatic acid), so there are five more ν_=CH_ within phenyl rings (3073 cm^−1^), which directly leads to a decrease in the relative peak intensities for ν_asCH2_ (2927 cm^−1^) and ν_asCH3_ (2959 cm^−1^) in the EB spectrum to 0.7 and 0.9, respectively. EV and EB could be identified by this feature. Furthermore, since there are two benzene rings in EB, the characteristic peaks from ν_C=C_ (1.3) and δ_-CH_ within phenyl rings (1.5) are stronger than those from EV. At the same time, a group of quadruple peaks that do not appear in EV appear at 1269~1164 cm^−1^ in the spectrum of EB, and the peak from ν_C=O_ in EB is stronger than that in EV. The difference in these characteristic peaks can also be utilized to distinguish EV from EB. The aromatic–CH stretch mode within the 3000–3100 cm^−1^ region is useful in discriminating EB from E3, E2, EV, CEE, and EE2 at a minimum. The EB spectra reveal aromatic–CH stretch modes at 3073 cm^−1^, while the others exhibit modes around 3060 cm^−1^. The slight difference in the peak position of the aromatic–CH stretch mode for the different estrogens can be attributed to the differences in the packing of the molecules as revealed by their respective crystal structures [27].

CEE and EE2 belonged to the third category. Their chemical structures do not contain a carbonyl group, but both contain an alkynyl group. A prominent peak at 2115 cm^−1^ on CEE and doublet peaks at 2113 and 2102 cm^−1^ on EE2 were observed arising from the C≡C stretching mode found of their ethylene group, which usually appear in the 2150–2100 cm^−1^ region [26] (P. 83). Moreover, for EE2, the peak from ν_asCH2_ (2924 cm^−1^) is significantly weaker than the peak from ν_asCH3_ (2941 cm^−1^), and the peak intensity ratio is 2.4/2.7 (value < 1). In the structure of CEE, the phenolic hydroxyl group is ether-formed with cyclopentane, so there are four more peaks from ν_CH2_ than in EE2. This results in significant increase in the peak strength from ν_asCH2_ in the CEE spectrum. The peak intensity ratio of the characteristic peaks from ν_asCH2_ (2933 cm^−1^) to ν_asCH3_ (2966 cm^−1^) is increased to 4.2/3.5 (value > 1). This can be used to distinguish CEE from EE2. In addition, cyclopentane ether in CEE may interfere with the Raman scattering from the out-of-plane bending vibration of the benzene ring and make the characteristic peak at 713 cm^−1^ of CEE almost disappear, while the peak intensity ratio of EE2 at this Raman shift is 1.4.

### 2.4. Simulated Positive Sample Test

**Figure 6:**
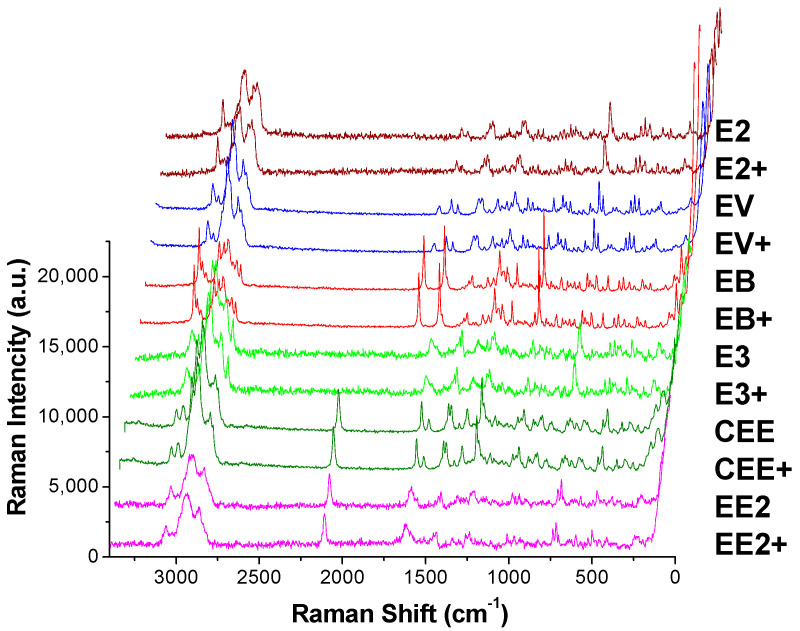
The Raman spectra of simulated positive pharmaceutical preparations based on TLC (E2, EV, EB, E3, CEE, and EE2: reference standard of the six estrogens; +: simulated positive sample).

Taking the simulated positive sample of EE2 tablets as an example, firstly, the negative sample solution and simulated positive sample solution were prepared, respectively. Then, the simulated positive sample and negative sample solution were spotted on the same thin-layer plate, and their Raman spectra were measured, respectively. In the same way, then, the Raman spectra of simulated positive preparations were detected. The results showed that no Raman signal was detected in the negative sample solutions of the various preparations. The simulated positive sample and its corresponding reference standard had the same main spot position on TLC. Moreover, the peak position, peak shape, and peak intensity of the Raman spectra were basically consistent, as shown in Figure 6. Therefore, the excipients in tablets, injections, and suppositories had little influence on the detection of the studied compounds by Raman spectroscopy.

### 2.5. Precision Inspection

The reference solutions of studied compounds were measured six times in total. The Raman shifts of the minimum characteristic peaks in the Raman spectra were found to be 1611 cm^−1^ _(_ν_C=C_), 1609 cm^−1^ _(_ν_C=C_), 1697 cm^−1^ _(_ν_C=O)_, 1435 cm^−1^ _(_δ_CH3,_ δ_CH2)_, 1616 cm^−1^ _(_ν_C=C)_, and 1601 cm^−1^ _(_ν_C=C)_, respectively. Based on the relative intensity of these peaks, the RSD of the six estrogens were calculated to be 1.21, 1.45, 1.56, 3.32, 0.97, and 2.01%. This indicates that the instrument has good repeatability.

### 2.6. Determination of the Limit of Detection (LOD)

**Figure 7:**
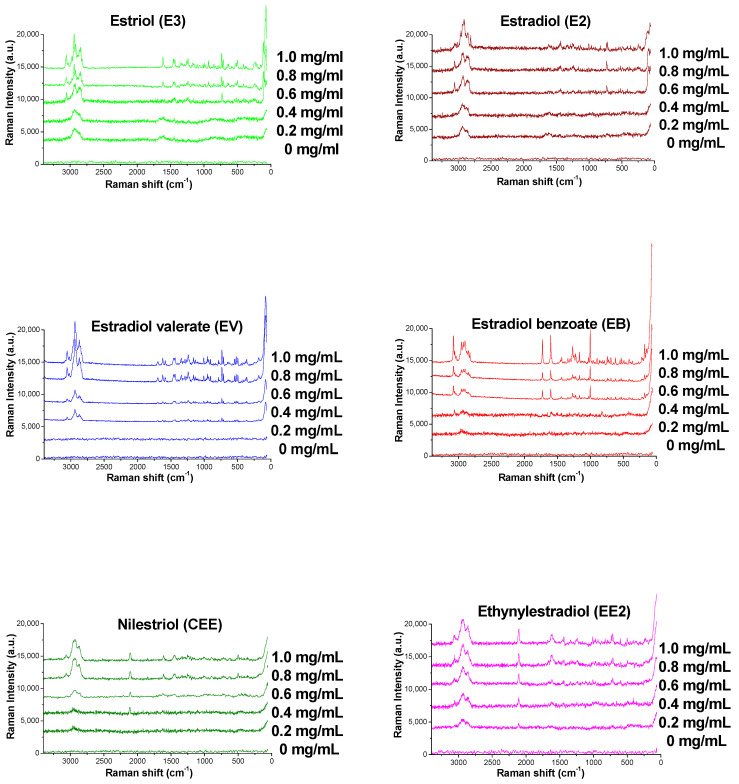
The Raman spectra of estrogens at different concentrations based on TLC.

**Figure 8:**
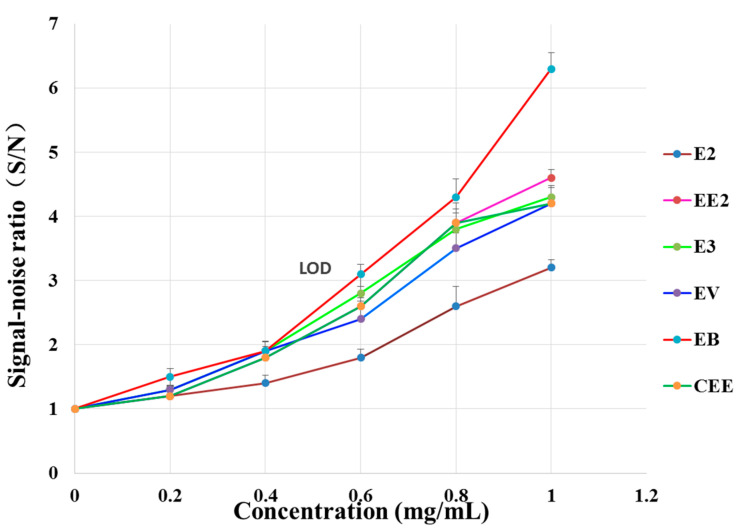
The LOD analysis of six reference substances.

Taking E2 as an example, a gradient solution of E2 was prepared by sequentially diluting the reference solution to concentrations of 0.200, 0.400, 0.600, 0.800, and 1.00 mg/mL. Similarly, the gradient solutions of E3, EV, EB, CEE, and EE2 were prepared. The Raman spectra of various solutions are presented in Figure 7. The signal-to-noise ratios (S/N) were calculated by characteristic peaks with Raman shifts at 1611 cm^−1^ _(_ν_C=C)_, 1609 cm^−1^ _(_ν_C=C)_, 1697 cm^−1^ _(_ν_C=O)_, 1435 cm^−1^ _(_δ_CH3,_ δ_CH2)_, 1616 cm^−1^ _(_ν_C=C)_, and 1601 cm^−1^ _(_ν_C=C)_, respectively. Based on the above operations, the S/N of each estrogen was plotted against its concentration (C), and a standard curve was drawn (see Figure 8). When concentration of each estrogen was more than 0.4, the signal-to-noise ratio was linearly to the concentration of the corresponding reference substances, the regression equations are Y(E3) = 4.1x + 0.33 (r = 0.9915), Y(E2) = 3.1x + 0.08 (r = 0.9928), Y(EV) = 4x + 0.2 (r = 0.9908), Y(EB) = 7.2x + 0.9818 (r = 0.9909), Y(CEE) = 4.25x + 0.9538 (r = 0.9766), and Y(EE2) = 4.85x + 0.17 (r = 0.9934), respectively. According to the regression equations, the corresponding estrogen concentrations when S/N = 3 were calculated, which represents the limit of detection (LOD) of the estrogens. These values are 0.636, 1.00, 0.687, 0.497, 0.649, and 0.626 mg/mL, respectively. These values can fulfill the analytical requirements of the test solution (1 mg/mL).

### 2.7. Stability Test

The reference solutions were prepared in accordance with 2.4. Subsequently, the Raman spectra of them were measured after being placed 10, 20, 40, and 60 min, respectively. And then, the relative peak intensities at 1611 cm^−1^ _(_ν_C=C)_, 1609 cm^−1^ _(_ν_C=C)_, 1697 cm^−1^ _(_ν_C=O)_, 1435 cm^−1^ _(_δ_CH3,_ δ_CH2)_, 1616 cm^−1^ _(_ν_C=C)_, and 1601 cm^−1^_(_ν_C=C)_ were calculated. The stabilities at different times were studied. The results indicated that the Raman spectra of six estrogens were stable within 40 min, with the RSD values of 1.34, 2.06, 1.65, 3.99, 1.16, and 2.71%, respectively.

### 2.8. Detection of Real Samples

**Figure 9:**
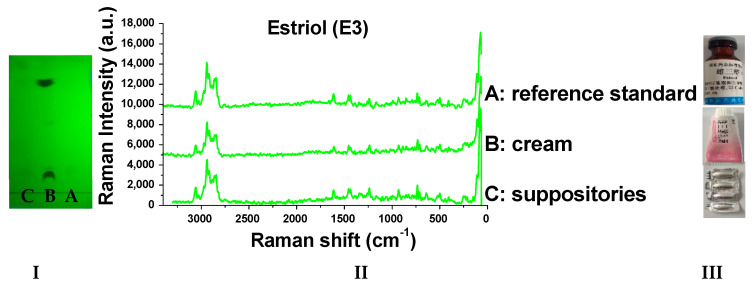
Detection results of estriol samples (**I**: TLC plate; **II**: Raman spectra; **III**: photos of drug; A: reference standard; B: cream; C: suppositories).

**Figure 10:**
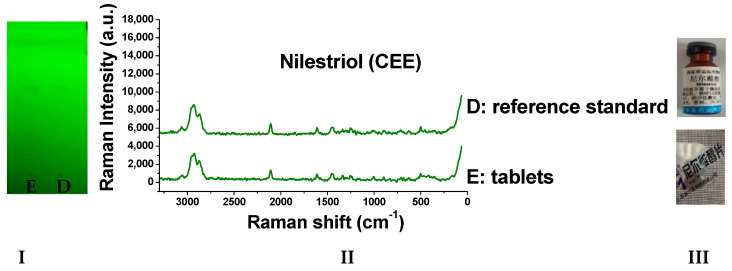
Detection results of nilestriol tablets (**I**: TLC plate; **II**: Raman spectra; **III**: photos of drug; D: reference standard; E: tablets).

**Figure 11:**
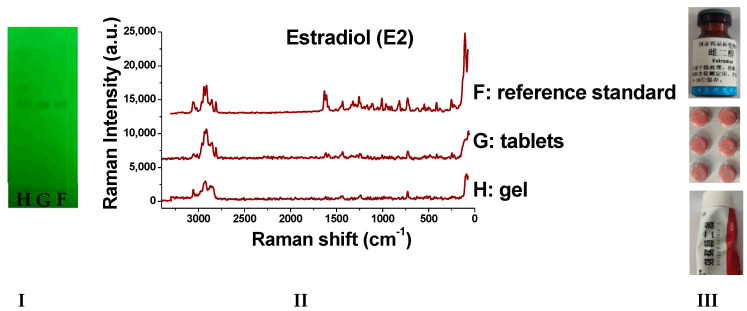
Detection results of estradiol samples (**I**: TLC plate; **II**: Raman spectra; **III**: photos of drug; F: reference standard; G: tablets; H: gel).

**Figure 12:**
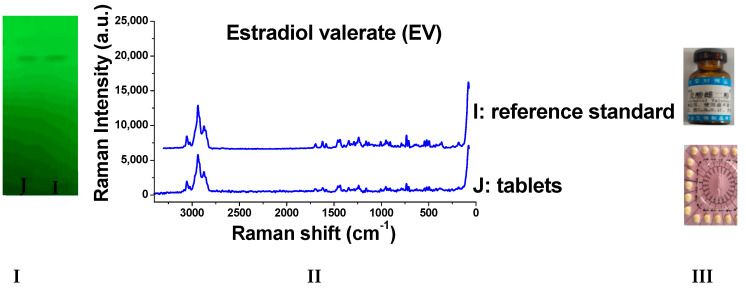
Detection results of estradiol valerate tablets (**I**: TLC plate; **II**: Raman spectra; **III**: photos of drug; I: reference standard; J: tablets).

**Figure 13:**
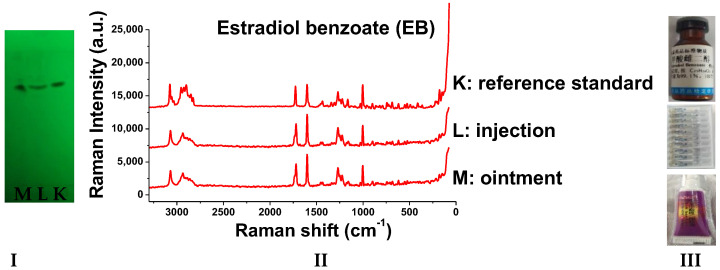
Detection results of estradiol benzoate samples (**I**: TLC plate; **II**: Raman spectra; **III**: photos of drug; K: reference standard; L: injection; M: ointment).

**Figure 14:**
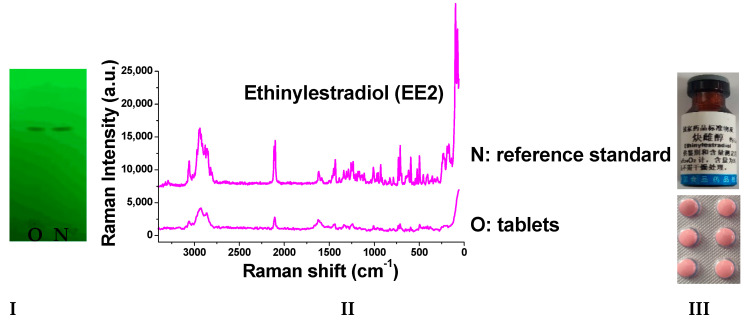
Detection results of ethinylestradiol tablets (**I**: TLC plate; **II**: Raman spectra; **III**: photos of drug; N: reference standard; O: tablets).

When the sample solutions were compared with the corresponding reference solutions, the results (Figure 9, Figure 10, Figure 11, Figure 12, Figure 13 and Figure 14) showed that the R_f_ values of the main spots on TLC were identical and the Raman spectra of the samples were the same as those of the references.

As we can see from the figures, there are other spots in some preparations (which might be due to other components in the drug or caused by the decomposition of the drug itself). Through the different R_f_ values on thin-layer chromatography, they can be easily distinguished. Moreover, the Raman spectrum measurement of spots with the same retardation factor further verifies the results. Based on this, this method can be used to identify whether there are substances caused by decomposition or counterfeiting in drugs.

## 3. Materials and Methods

### 3.1. Materials

E3, E2, EV, EB, CEE and EE2 reference standards were all purchased from China Institute of Food and Drug Control. Estradiol tablets (2 mg/tablet), estradiol valerate tablets (1 mg/tablet), estradiol benzoate injections (2 mg/1 mL), estriol suppositories (0.5 mg/piece), ethinylestradiol and cyproterone acetate tablets (each tablet contains ethinylestradiol 0.035 mg, cyproterone acetate 2 mg), and nilestriol tablets (1 mg/tablet) were all commercially available drugs.

Ethanol absolute (analytical grade, Tianjin Fuchen Chemical Reagent Factory, Tianjin, China), ethyl ether (analytical grade, Tianjin Jindong Tianzheng Fine Chemical Reagent Factory, Tianjin, China), acetic acid (analytical grade, Shanghai Miting Chemical Reagent Co., Ltd. Shanghai, China), and benzene (analytical grade Pure, Tianjin Fuchen Chemical Reagent Factory, Tianjin, China) were commercially available products.

### 3.2. Apparatus

An analytical balance with a precision of 0.01 mg (made in Switzerland) was used to weigh the reference standard. After that, the UP 50 ultrasonic cleaner (produced by Nanjing Leijunda Ultrasonic Electronic Equipment Co., Ltd. Nanjing, China) was employed to accelerate the dissolution process. For thin-layer chromatography (TLC), a chromatography cylinder (10 cm × 5 cm × 10 cm, manufactured by Shanghai Titan Technology Co., Ltd. Shanghai, China), TLC Silica gel 60 F_254_ Aluminum sheets (cut to 10 cm×10 cm before use, supplied by Merck KGaA, Darmstadt, Germany), and a quantitative capillary (2 μL, from Tianjin Silida Technology Co., Ltd. Tianjin, China) were used.

The compounds separated on TLC were located under 254 nm ultraviolet light using an ultraviolet analyzer (WFH-203B; Shanghai Jingke Industrial Co., Ltd. Shanghai, China). Raman spectra and their imaging were obtained by a DXR™ xi Raman Imaging Microscope (Thermo Fisher Scientific, Waltham, USA) with an excitation wavelength of 532 nm, a resolution of 5.0 cm^−1^, and a 10× long-working-distance microscope objective. The excitation power was 10.0 mW, the integration time was 0.5 s, and the number of scans was 20. The scan range was from 3300 cm^−1^ to 100 cm^−1^, with a 50 µm confocal pinhole DXR532 full range grating (400 line/mm). The detector was a TE-cooled electron-multiplying CCD (EMCCD). Area scanning was chosen as the scanning mode, the scanning area was more than 150 μm × 150 μm, and the total scanning time was 20 min.

### 3.3. Solutions Preparation

#### 3.3.1. Reference Solution

Appropriate amount of ethanol was added to the appropriate amounts of estrogen reference standards, respectively, to make solutions with a concentration of 1 mg/mL for each reference standard.

#### 3.3.2. Mixed Reference Solution

Appropriate amounts of estrogen reference standards (E2, EV, EB, E3, CEE, and EE2) were placed in the same container. Then, an appropriate amount of ethanol was added to ensure that the concentrations in the final solution were all 1 mg/mL.

#### 3.3.3. Sample Solution

Estradiol tablets, estradiol valerate tablets, ethinylestradiol tablets, and nilestriol tablets were ground into powder, respectively. Appropriate amounts of ethanol were added to the respective appropriate amounts of powders (approximately equivalent to 2 mg of E2, EV, EE2, and CEE). Then, ultrasonication was performed. The supernatant was filtered using a 0.22 μm disposable filter, and the filtrate was evaporated and redissolved with ethanol to prepare solutions containing approximately 1 mg/mL of E2, EV, EE2, and CEE, respectively.

Take an appropriate quantity of estradiol gel and place it in a centrifuge tube. Subsequently, add a suitable amount of absolute ethanol to the tube. Then, subject the mixture to ultrasonic treatment for 30 min. After that, centrifuge the sample and collect the supernatant. The concentration of the supernatant is equivalent to that of an ethanol solution with a concentration of 1.0 mg/mL.

An appropriate quantity of estradiol benzoate injections (approximately equivalent to 2 mg of EB) was vigorously mixed with 2 mL of ethanol. The solution was then placed in a water bath. After static layering, the ethanol solution from the upper layer was taken out and placed in a centrifuge tube. The tube was centrifuged, and the supernatant was taken as the sample solution with a concentration equivalent to 1 mg/mL of EB. For estradiol benzoate ointment, an appropriate amount was taken and placed in a centrifuge tube. An appropriate amount of absolute ethanol was added to the tube. The mixture was ultrasonicated for 30 min and then was centrifuged. The supernatant was taken and placed in a water bath to evaporate the solvent completely. The residue was dissolved with ethanol to obtain a solution with a concentration equivalent to 1.0 mg/mL of EB.

An appropriate quantity of estriol suppositories (approximately equivalent to 1 mg of E3) was placed in a conical flask. Subsequently, 10 mL of absolute alcohol was added, and the mixture was heated on a 60 °C water bath. The mixture was stirred until it was completely dissolved. After cooling to room temperature, the mixture was shaken thoroughly. When the substrate turns semi-solid at a temperature range of 5~10 °C, the filtration operation was carried out immediately. The filtrate was concentrated to 1 mL to serve as the sample solution with a concentration equivalent to 1 mg/mL of E3. Take an appropriate amount of estriol cream (equivalent to 1 mg of E3) and put it in a 10 mL beaker. Subsequently, 10 mL of absolute ethanol was added to the beaker. The beaker was placed in a water bath at 60 °C for heating. The mixture was stirred with a glass rod until it completely dissolved. The solution was cooled to room temperature, filtered, and the subsequent filtrate was taken. The filtrate was concentrated to 1 mL to obtain the test solution. The concentration of the test solution was equivalent to that of 1.0 mg/mL of E3.

#### 3.3.4. Negative Sample Solution

Self-made blank starch tablets, blank injections, and blank suppositories were used as negative samples. The negative sample solution was prepared following the method described in Section 3.3.3.

#### 3.3.5. Simulated Positive Sample Solution

Appropriate quantities of different types of self-made blank preparations (powder for tablets) were taken. Appropriate amounts of reference standards were added to prepare simulated positive estradiol tablets (2 mg per tablet), estradiol valerate tablets (1 mg per tablet), ethinyl estradiol tablets (each tablet contains 0.035 mg of ethinyl estradiol), nilestriol tablets (1 mg per tablet), estradiol benzoate injections (1 mg per 1 mL), and estriol suppositories (0.5 mg per piece). The simulated positive sample solution was prepared according to the method described in Section 3.3.3.

### 3.4. TLC Detection Method

Thin-layer chromatography (TLC) is a simple and rapid separation technique. In our study, 10 μL of six estrogen reference standards and their mixed solutions were spotted on a GF_254_ thin-layer plate (8 cm × 10 cm), 1 cm away from the bottom. Benzene–ether–glacial acetic acid (50:30:0.5, *v*/*v*/*v*) was used as the developing agent. After development, the plate was taken out, dried, inspected, and labeled under UV light (254 nm). All the R_f_ values of the estrogens were accurately measured.

### 3.5. Micro-Raman Imaging Spectroscopy of Estrogens Based on TLC Spots

After in situ enrichment of the samples, the labeled spots on the plate were measured by Raman spectroscopy. This enrichment process increased the molecular concentration and improved the detection sensitivity. Then, the plate was placed directly under a microscope. A 532 nm laser was used as the light source to pass through the area. The Raman scattering image of the component to be measured was obtained by surface scanning. Based on the distribution of the components to be measured in the image, the Raman spectrum was directly obtained.

### 3.6. Determination of Samples

Ten microliters of the sample solution and the corresponding reference solution were spotted, respectively, on the silica gel GF_254_ thin-layer plate. After development, the plate was taken out, dried, inspected, and labeled under UV light (254 nm). The R_f_ values of all the estrogens were accurately measured. All the components to be tested were enriched in situ with ethanol, and the Raman spectra of the component to be tested were obtained through the Raman image. The R_f_ values and Raman spectra of the sample spots should be consistent with those of the corresponding reference.

## 4. Conclusions

Steroid estrogens are a series of homologues sharing the same steroid nucleus yet having different substituents. Some drugs within this group differ from each other only by a single hydroxyl group or methyl group, which restricts the specificity of identification when using infrared spectroscopy (IR) or chromatography alone. Consequently, in CHP2020, both IR and chromatography were utilized to identify the drug substances of the same steroid estrogens, significantly enhancing specificity. Nevertheless, for various preparations of steroid estrogens, the situation is quite different. When using IR to identify steroid estrogen preparations, it is usually necessary to extract the active ingredients from excipients to obtain solid compounds with a purity higher than 90%. Meanwhile, to avoid the differences between the infrared spectrum and the standard spectrum (Atlas of infrared spectra of drugs) caused by the crystal form transformation of the drug during the extraction process, it is also essential to conduct parallel experiments with a substantial amount of reference material (about 50–100 mg). These pretreatment processes are not only time-consuming and tedious, but also costly; therefore, IR is not employed to identify the preparations of steroid estrogens in CHP2020, and HPLC is adopted instead. However, the specificity of this method is lower than that of drug substance identification.

Raman scattering spectroscopy is similar to infrared spectroscopy in terms of specificity as both can reflect the abundant structural information of compounds. Hence, Raman spectroscopy can also function as a more specific identification technology. The study established a combined technology of TLC and Raman scattering spectroscopy for the identification of estrogen preparations. This method entails extraction and purification by an appropriate pretreatment method, followed by thin-layer chromatography separation and Raman imaging detection. The different R_f_ values of drugs on TLC and the unique Raman spectral characteristics, respectively, mirror the polarity and structural differences of drugs, enabling accurate distinction of six estrogens. This method is free from interference by excipients in preparations and has the advantages of high specificity, good stability, and high sensitivity.

In conclusion, this study provides a reliable and effective new approach for the identification of six estrogen preparations. The specificity of this method is higher than that described in the ChP2020, and there is no need to extract high-purity solid components from the preparations. This method is anticipated to be further applied to the identification of numerous other drug preparations.

## Figures and Tables

**Table 1:** The comparison of Raman spectra characteristic peaks between enrichment on TLC and reference standard of six estrogens.

Compounds and Structures	Raman Shifts of Reference Standard/cm^−1^ (Relative Peak Intensity)	Raman Shifts of Spectra by TLC-RIM/cm^−1^ (Relative Peak Intensity)	Assignments
Estriol (E3) 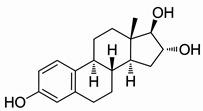	3060 (1.0) 2940, 2913 (2.4, 1.7) 2845, 2823 (1.5, 0.7) 1614 (Singlet: 0.9) 1460~1426 (Triplet: 0.7~0.2) 1245 (0.7) 735, 721 (1.2, 0.7)	3059 (1.0) 2940, 2919 (2.4, 1.8) 2843, 2825 (1.6, 0.8) 1611 (Singlet: 0.7) 1456~1422 (Triplet: 0.8~0.7) 1246 (0.7) 732, 722 (1.2, 0.7)	ν_=CH within phenyl rings_ ν_asCH3,_ ν_asCH2_ ν_sCH3,_ ν_sCH2_ ν_C=C_ δ_-CH3,_ δ_-CH2_ ν_c-c (ring stretch shift)_ γ_=C-H within phenyl rings_
Estradiol (E2) 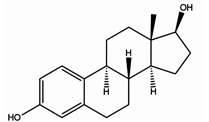	3060 (1.0) 2939, 2913 (3.9, 4.2) 2858, 2815 (2.5, 2.0) 1619, 1612 (0.7, 0.6) 1457~1423 (Triplet: 0.9~0.5) 1261 (0.9) 732, 725 (1.2, 0.6)	3060 (1.0) 2936, 2915 (3.6, 3.8) 2855, 2813 (1.9, 1.4) 1622, 1609 (0.7, 0.5) 1456~1422 (Triplet: 0.9~0.5) 1264 (0.8) 732, 722 (1.0, 0.5)	ν_=CH within phenyl rings_ ν_asCH3,_ ν_asCH2_ ν_sCH3,_ ν_sCH2_ ν_C=C _ δ_CH3,_ δ_CH2_ ν_c-c (ring stretch shift)_ γ_=C-H within phenyl rings_
Estradiol valerate (EV) 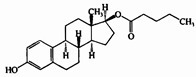	3057 (1.0) 2937, 2921 (3.2, 2.3) 2874, 2857 (2.3, 1.7) 1697 (0.4) 1621 (0.7) 1444 (0.9) 1243 (0.9) 734, 710 (1.2, 0.7)	3056 (1.0) 2938, 2925 (3.1, 2.4) 2874, 2855 (1.8, 1.3) 1697 (0.5) 1620 (0.6) 1445 (0.6) 1241 (0.6) 734, 710 (1.0, 0.7)	ν_=CH within phenyl rings_ ν_asCH3,_ ν_asCH2_ ν_sCH3,_ ν_sCH2_ ν_C=O _ ν_C=C_ δ_CH3,_ δ_CH2_ ν_c-c (ring stretch shift)_ γ_=C-H within phenyl rings_
Estradiol benzoate (EB) 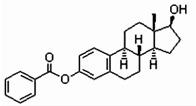	3073 (1.0) 2959, 2927 (0.9, 0.7) 2854, 2829 (0.6, 0.5) 1727 (1.1) 1603 (1.3) 1432 (0.2) 1269–1164 (Quartet: 1.1~0.2) 1009 (1.5)	3069 (1.0) 2956, 2928 (0.9, 0.7) 2851, 2830 (0.6, 0.5) 1721 (1.3) 1608 (1.9) 1435 (0.2) 1274~1169 (Quartet: 1.1~0.2) 1004 (1.5)	ν_=CH within phenyl rings_ ν_asCH3,_ ν_asCH2_ ν_sCH3,_ ν_sCH2_ ν_C=O_ ν_C=C_ δ_CH3,_ δ_CH2_ ν_c-c (ring stretch shift)_ δ_-CH within phenyl rings_
Nilestriol (CEE) 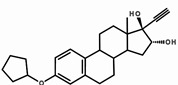	3054 (1.0) 2966, 2933 (3.5, 4.2) 2860, 2845 (2.1, 1.7) 2115 (1.7) 1618, 1574 (Doublet: 1.3, 0.6) 1452~1441 (Doublet: 0.9, 0.8) 1261~1242(Doublet: 1.0, 0.7)	3058(1.0) 2962, 2930 (3.5, 4.2) 2863, 2850 (2.2, 2.2) 2113 (1.3) 1616 (Singlet: 0.8) 1454~1441 (Doublet: 1.0, 0.8) 1259~1247 (Doublet: 1.0, 1.0)	ν_=CH within phenyl rings_ ν_asCH3,_ ν_asCH2_ ν_sCH3,_ ν_sCH2_ ν_C≡C_ ν_C=C_ δ_CH3,_ δ_CH2_ ν_c-c (ring stretch shift)_
Ethinylestradiol (EE2) 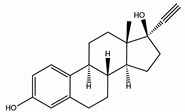	3058(1.0) 2941, 2924 (2.7, 2.4) 2868, 2860 (1.6, 1.7) 2113, 2102 (Doublet: 1.4, 1.5) 1617, 1602 (Doublet: 0.7, 0.5) 1439 (Doublet: 0.7, 0.9) 1241 (Doublet: 0.8, 0.9) 735, 713 (1.1, 1.4)	3061 (1.0) 2937, 2919 (3.0, 2.3) 2870, 2864 (2.0, 2.4) 2111 (Singlet: 1.7) 1615, 1601 (Doublet: 0.6, 0.5) 1452~1437 (Doublet: 0.7, 0.9) 1259~1244 (Doublet: 0.7, 1.1) 733, 716 (1.0, 1.4)	ν_=CH within phenyl rings_ ν_asCH3,_ ν_asCH2_ ν_sCH3,_ ν_sCH2_ ν_C≡C_ ν_C=C_ δ_CH3,_ δ_CH2_ ν_c-c (ring stretch shift)_ γ_=C-H within phenyl rings_

Note: ν: stretching vibration; γ: out-of-plane bending; δ: in-plane bending. The red words represent the difference in the number or intensity of Raman characteristic peaks after the drug is separated on TLC plate, as compared with the corresponding reference standard.

## Data Availability

The original contributions presented in the study are included in the article, further inquiries can be directed to the corresponding author.
